# Hematometra: A Rare Case of Pelvic Pain in Females Identified with Point of Care Ultrasound

**DOI:** 10.24908/pocus.v9i1.16988

**Published:** 2024-04-22

**Authors:** Andrew R Helber, Margaret Provencher, Christy Moore, Nova Panebianco

**Affiliations:** 1 Department of Emergency Medicine, University of Pennsylvania Philadelphia, PA USA

**Keywords:** Point of Care Ultrasound, Hematometra, Ultrasound Case Presentation

## Abstract

The differential diagnosis for abdominal or pelvic pain in women of child-bearing age that present to the emergency department is broad. A rare cause of abdominal and pelvic pain is hematometra, or a collection of blood products within the uterus. While blood is normally expelled through menses, this process is disrupted in some patients due to congenital or acquired abnormalities. This can lead to progressive uterine distension and pain, which may ultimately require medical or surgical intervention. Hematometra is rare, but is a serious condition that can be diagnosed easily at bedside using point of care ultrasound.

## Introduction

An 18-year-old woman with history of unrepaired cervico-vaginal atresia presented to the emergency department (ED) with progressive, diffuse abdominal pain and distension. This patient recently immigrated to the United States and had not yet established gynecologic care. She noted that she had previously been on estrogen therapy to prevent menses but no longer had access to this prescription medication.

On arrival, the patient was distressed due to pain and had a distended and firm lower abdomen that appeared gravid. She had stable vital signs and the remaining physical exam was unremarkable. Her urine pregnancy test was negative and the first imaging performed was a pelvic point of care ultrasound (POCUS) examination by the ED team. The differential diagnosis included hemorrhagic ovarian cyst, ovarian torsion, tubo-ovarian abscess, pelvic-inflammatory disease, urinary retention, and hematometra (collection of blood within the uterine cavity), among other intrabdominal pathology.

**Figure 1:**
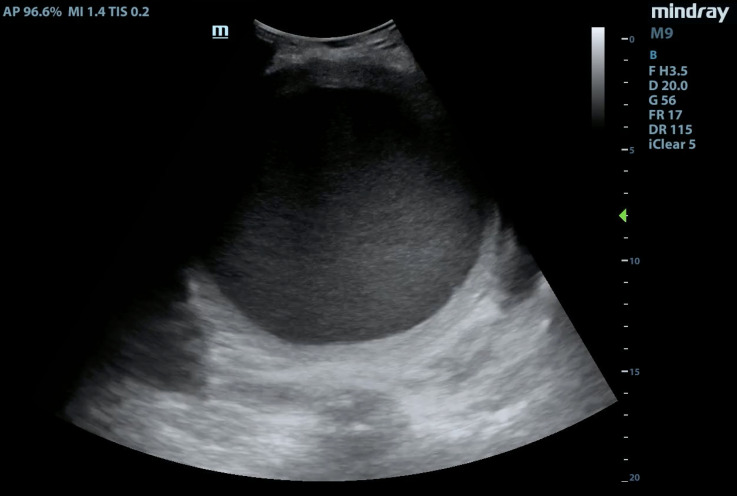
Point of Care Ultrasound Demonstrating an Enlarged Uterus Full of Hypoechoic Fluid in the Transverse Plane.

**Figure 2:**
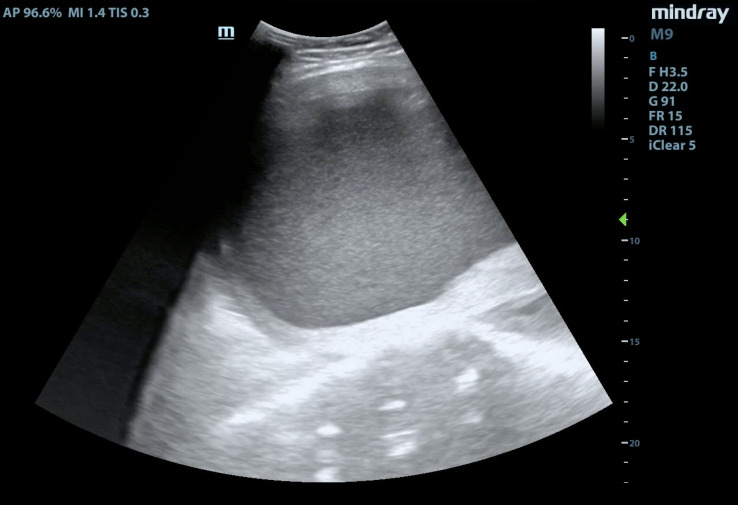
Point of Care Ultrasound Demonstrating an Enlarged Uterus Full of Hypoechoic Fluid in the Longitudinal Plane.

**Figure 3:**
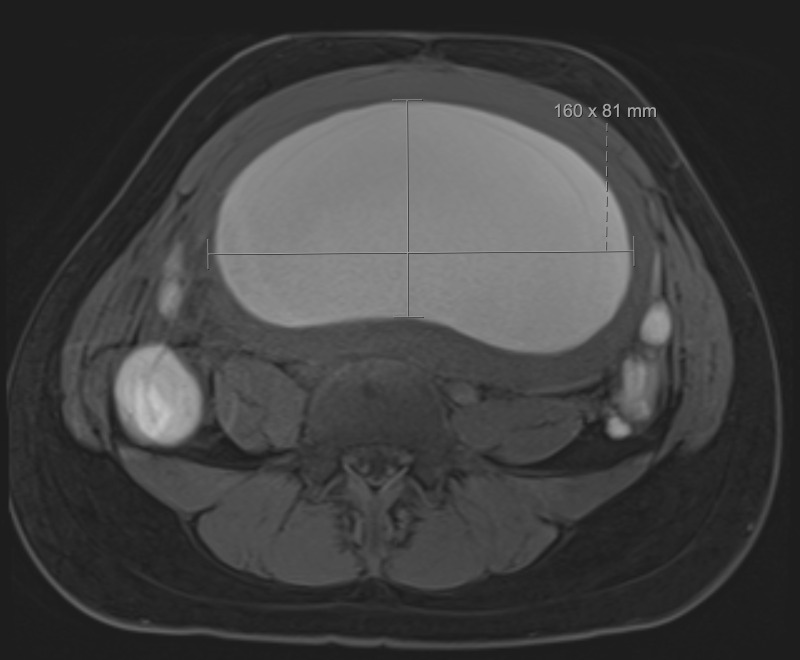
T1 Weighted Axial MRI Image.

**Figure 4:**
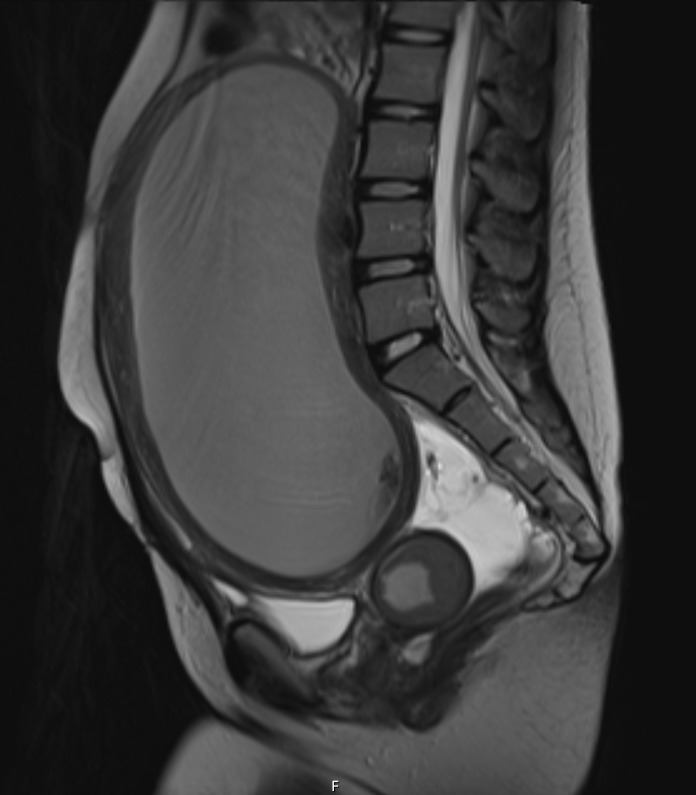
T2 Weighted Sagittal MRI Image.

A focused assessment for free fluid (FAFF) exam was performed. It was negative for intra-abdominal free fluid, however, it was notable for a distended uterus above the level of the umbilicus that was filled with homogeneous, hypoechoic material – presumed to be blood (Figure 1, 2). The patient was treated with analgesics and gynecology was consulted. She was admitted and ultimately received a pelvic MRI for surgical planning, which confirmed the findings of a hematometra (Figure 3, 4). Interventional radiology was consulted and a percutaneous uterine drain was placed, which drained her hematometra and relieved her abdominal distension and pelvic pain. The patient was discharged the next day with plans to schedule outpatient surgery for definitive reconstruction. Notably, no radiologic study with ionizing radiation was required in the work up of this patient by utilizing a POCUS-first strategy. 

## Differential Diagnosis and Imaging Strategy

While this patient had known cervico-vaginal atresia which brought hematometra to the top of the differential, in a different scenario the diagnosis could be more challenging. Choosing a POCUS-first approach could expedite the gynecologic consult and obviate the need for additional imaging. The treating team chose to perform a FAFF exam which looks specifically for intra-abdominal free fluid; however, because the protocol examines a broad anatomic region, abnormalities other than free fluid may be encountered. POCUS for obstetric or gynecologic pathology would also have been appropriate, which could include transabdominal and transvaginal imaging. Knowledge of hematometra and other pelvic pathologies, as well as the use of POCUS as an imaging modality in their evaluation, is relevant to multiple specialties including pediatrics, pediatric emergency medicine, adult emergency medicine, gynecology, and radiology. Hematometra can be caused by several etiologies that affect multiple age groups, including adolescents with an imperforate hymen and older patients that develop cervical outlet obstructions secondary to tumors, post-surgical scaring, post-radiation complications, or foreign bodies. Hematometra should be on the differential for most women with lower abdominal pain.

It is important to scan intentionally and identify anchoring anatomy so that when pathology is encountered it is recognized. In the pelvis, the bladder is usually a fluid-filled structure. The uterus can be differentiated from the urinary bladder by its relative location to the pubic symphysis and by (on transabdominal imaging) tracking the vaginal stripe to the cervix/lower uterine segment. Usually, the uterus has thick walls and has minimal intrauterine fluid compared to the bladder, which has thinner walls and is filled with anechoic fluid.

## Conclusion

In this case we describe a typical physical exam and ultrasound findings in a rare ED case of hematometra. A POCUS-first imaging strategy prevented exposing this woman of child-bearing age to ionizing radiation and expedited care and expert consultation. Additionally, it exposed the vulnerability of patients who do not have access to out-patient care and medications. Knowledge of the condition and sonographic findings is relevant to any physician who cares for women of child-bearing age. 

## Disclosures

No disclosures.

## Patient Consent

The patient consented to use of deidentified photos, videos, and recordings for education and research purposes.

## Supplementary Material

 Video S1 Point of care ultrasound demonstrating an enlarged uterus full of hypoechoic fluid in the transverse plane.

Video S2 Point of care ultrasound demonstrating an enlarged uterus full of hypoechoic fluid in the longitudinal plane.

